# When Chirality Makes the Difference: The Case of Novel Enantiopure *N*-Heterocyclic Carbene–Gold and –Silver Complexes

**DOI:** 10.3390/molecules29225262

**Published:** 2024-11-07

**Authors:** Maria Marra, Annaluisa Mariconda, Domenico Iacopetta, Jessica Ceramella, Assunta D’Amato, Camillo Rosano, Kateryna Tkachenko, Michele Pellegrino, Stefano Aquaro, Maria Stefania Sinicropi, Pasquale Longo

**Affiliations:** 1 Department of Pharmacy, Health and Nutritional Sciences, University of Calabria, Via P. Bucci, 87036 Arcavacata di Rende, Italy; mariamarra1997@gmail.com (M.M.); domenico.iacopetta@unical.it (D.I.); michele.pellegrino@unical.it (M.P.); s.sinicropi@unical.it (M.S.S.); 2 Department of Science, University of Basilicata, Viale dell’Ateneo Lucano 10, 85100 Potenza, Italy; annaluisa.mariconda@unibas.it; 3 Department of Chemistry and Biology, University of Salerno, Via Giovanni Paolo II, 132, 84084 Fisciano, Italy; asdamato@unisa.it (A.D.); plongo@unisa.it (P.L.); 4 U.O. Proteomica e Spettrometria di Massa, IRCCS Ospedale Policlinico San Martino, L.go Rosanna Benzi 10, 16132 Genova, Italy; kateryna.tkachenko@hsanmartino.it; 5 Department of Life, Health and Environmental Sciences, University of L’Aquila, Piazzale Salvatore Tommasi 1, Blocco 11, Coppito, 67010 L’Aquila, Italy; stefano.aquaro@univaq.it

**Keywords:** *N*-heterocyclic carbenes (NHC), enantiopure complexes, iNOS inhibition, anticancer, antibacterial

## Abstract

*N*-heterocyclic carbene (NHC)–gold and –silver complexes have attracted the interest of the scientific community because of their multiple applications and their versatility in being chemically modified in order to improve their biological properties. However, most of these complexes contain one or more chiral centers, and have been obtained and studied as racemic mixture. In particular, concerning the interesting biological and medicinal properties, many questions about how the chirality may influence these properties still remain unanswered. Aiming at a better understanding, herein a series of enantiopure NHC–gold and –silver complexes was synthesized, characterized and biologically evaluated in different in vitro systems. The individuated complexes exerted different properties based on the complexed metal and the specific configuration, with the (*R*)-gold–NHC complexes being the most active, particularly as anti-inflammatory molecules. Docking simulations indicated a different binding mode for each enantiomer. Moreover, anticancer and antibacterial activities were also evaluated for the considered enantiomers. Overall, the reported data may contribute to a better understanding of the different biological properties exerted by the enantiopure gold and silver complexes.

## 1. Introduction

**Figure 1:**
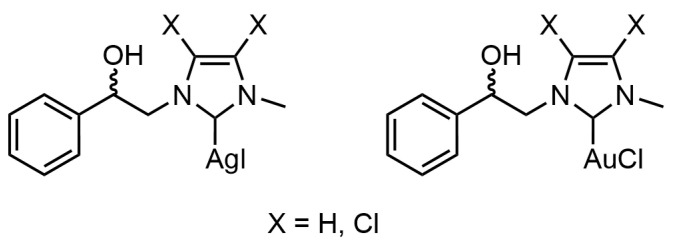
Anticancer Ag(I)– and Au(I)–NHC complexes previously reported [13,14].

Transition metal complexes have been demonstrated as a fruitful source in drug development. Particularly, those stabilized by carbenes ligands, which are versatile scaffolds for various structural modifications, have been demonstrated to target important biomolecular structures implied in different cellular pathways [1]. Moreover, these complexes have attracted considerable research interest due to numerous applications not only in medicinal chemistry but also in materials science and catalysis [2,3,4]. For instance, complexes based on ruthenium, copper, silver and gold with *N*-heterocyclic carbene (NHC) ligands frequently revealed significant antitumor activity [5,6,7,8]. Silver–NHC complexes have been proven to be important antimicrobial agents, since the silver cation interacts with the membrane or with the thiol groups of bacterial enzymes; they have also shown an interesting antiproliferative activity on cancer cell lines [9,10,11,12,13]. Last, but not least, gold–NHC complexes have been tested for their anti-arthritis, anti-inflammatory and anticancer properties showing, in the latter case, multiple mechanisms of action. Among these is the induction of mitochondrial or DNA damage, the regulation of various enzymes (e.g., thioredoxin reductase, kinases, phosphatases, topoisomerases) and the interference with cell cytoskeleton dynamics, which trigger cellular apoptosis [14,15,16,17,18,19]. Recently our research group obtained interesting results regarding the anticancer properties of silver– and gold–NHC complexes toward different cell lines. The activity of gold complexes against the breast cancer cell lines MCF-7 and MDA-MB-231 was particularly high, with IC_50_ values in the micromolar range [13,14]. The class of synthesized complexes possessed an NHC ligand having a stereogenic center (Figure 1) and they were tested as a racemate. However, the resolution of racemic mixtures has become fundamental in modern medicine for the development of new, more effective and safe drugs, because, as it is known, the stereoisomers often possess notable differences in pharmacodynamic, pharmacokinetic and toxicological properties.

Although selectivity is critical for the development of more effective drugs, to date, only few data comparing the different biological activity of enantiopure organometallic complexes have been reported [20,21]. Aiming at contributing to this interesting argument, in this work we separately synthesized some NHC–silver and –gold complexes with opposite chirality and evaluated their activity, in order to verify whether the *R* and *S* enantiomers could possess different biological profiles. Particularly, we focused our attention to their anti-inflammatory, anticancer and antibacterial properties. The anti-inflammatory activity was estimated by measuring the ability in decreasing the NO production in LPS-stimulated RAW 264.7 macrophages. Our outcomes indicate that only the (*R*)-NHC–gold complexes possessed anti-inflammatory activity. In silico studies performed on iNOS furnished some insights for the observed activity. Next, the anticancer properties were evaluated against two breast cancer cell lines, the ER-α–positive MCF-7 and the triple-negative MDA-MB 231 cell lines. The human mammary epithelial cells, MCF-10A, were also used as a control. The obtained IC_50_ values clearly indicate that, in most cases, just one enantiomer possesses a higher activity and selectivity. Finally, almost all the complexes show a fair antibacterial activity, particularly the NHC–silver complexes against two bacterial strains. The obtained outcomes may contribute to current knowledge on the use of enantiopure gold- and silver-based NHC complexes, strengthening the need to identify the eutomer for the development of new multitarget enantiopure pharmacological agents.

## 2. Results and Discussion

**Scheme 1:**
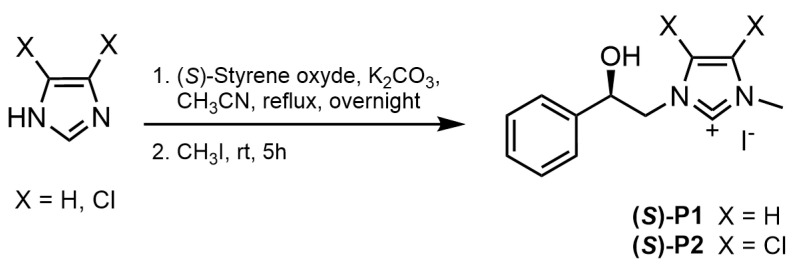
Synthesis of enantiopure pro-ligands **(*S*)-P1** and **(*S*)-P2**.

The synthesis of the enantiopure (*S*) complexes was carried out by reacting imidazole or 4,5-dchloro-imidazole with (*S*)-(−)-styrene oxide, in the presence of K_2_CO_3_. This base deprotonates the amino-nitrogen of imidazole producing the nucleophilic species. The nucleophilic attack of this nitrogen on the less substituted carbon of the epoxide ring causes its opening, leading to the formation of a β-amino alcohol. Since the opening of the oxirane ring is always trans, this reaction produces *N*-((*S*)-2-hydroxy-2-phenyl-ethyl)-imidazole or *N*-((*S*)-2-hydroxy-2-phenyl-ethyl)-4,5-dichloro-imidazole, respectively. The following step involves the reaction between these products with methyl iodide to give the alkylation of the sp^2^-hybridized nitrogen atom producing the iodo [*N*-methyl, *N*’-((*S*)-2-hydroxy-2-phenyl)ethyl-imidazol-2-ylidine] (**(*S*)-P1**) or iodo [4,5-dichloro *N*-methyl, *N*’-((*S*)-2-hydroxy-2-phenyl) ethyl-imidazol-2-ylidine] pro-ligands (**(*S*)-P2**) (see Scheme 1).

Similarly, to obtain pro-ligands with the opposite absolute configuration of the asymmetric carbon atom, it is necessary to use (*R*)-(+)-styrene oxide as an oxirane reagent. ^1^H- and ^13^C-NMR, mass spectroscopy and elemental analysis confirmed the structure of the salts. A polarimetric analysis allowed us to evaluate the specific rotation (${\left[\alpha \right]}_{D}^{25}$) of the compounds. The values obtained, reported in Table 1, are consistent with what was expected.

**Scheme 2:**
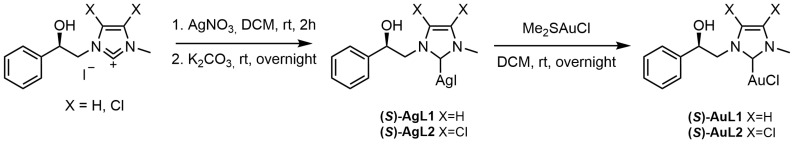
Synthesis of **(*S*)-AgL1**, **(*S*)-AgL2**, **(*S*)-AuL1** and **(*S*)-AuL2.**

The synthesis of NHC–metal complexes was carried out following the procedure previously reported in the literature [13,22] and shown in Scheme 2. To the pro-ligands **(*S*)-P1** and **(*S*)-P2**, AgNO_3_ is added and afterward potassium carbonate, which generates the respective carbene ligands **(*S*)-L1** and **(*S*)-L2** in situ. These undergo metalation by Ag(I) to generate the **(*S*)-AgL1** and **(*S*)-AgL2** complexes. Lastly, transmetalation with Me_2_SAuCl produces the analogous gold complexes **(*S*)-AuL1** and **(*S*)-AuL2**.

The complexes were characterized by ^1^H- and ^13^C-NMR, mass spectroscopy and elemental analysis.

Obviously, the data obtained are exactly the same as those found with the racemic complexes and already previously reported. The reactions reported in Scheme 2 cannot lead to racemization of our chiral center; however, to confirm this assumption, we also determined the specific rotation of the pair of complexes **(*S*)-AgL1**, **(*R*)-AgL1** and **(*S*)-AuL1**, **(*R*)-AuL1**, which have the chiral center with the opposite absolute configuration. The data obtained are consistent with what was hypothesized (see Table 1). Moreover, circular dichroism spectra of the final enantiomeric complexes (***S*)-AuL1** and **(*R*)-AuL1** showed to be superimposable and opposite (see Supporting Information, Figure S1).

### 2.1. Nitric Oxide (NO) Synthesis Inhibition

**Figure 2:**
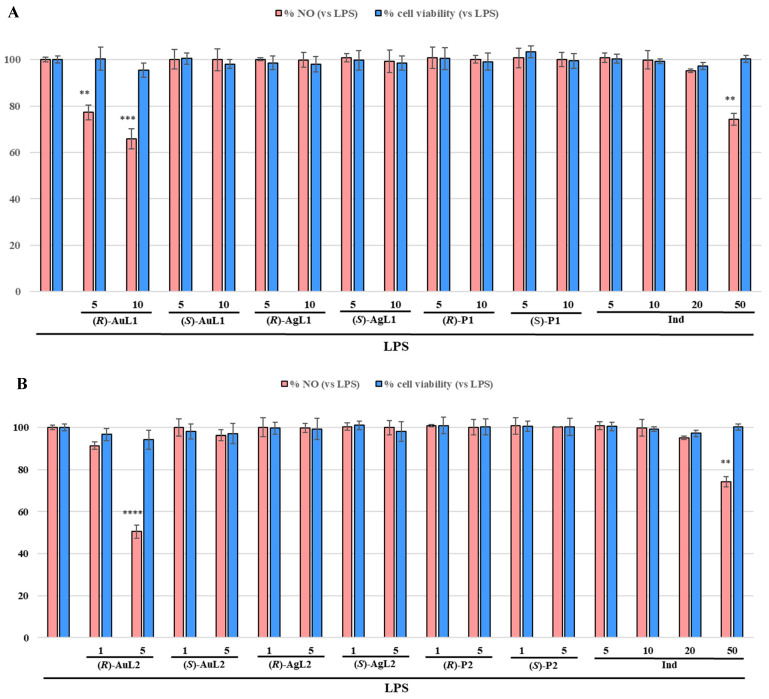
Panel (**A**), **L1** series and panel (**B**), **L2** series. Anti-inflammatory activity (pink bars): NO production was induced in murine macrophages RAW 264.7 treated with LPS (1 µg/mL), in presence or not of the enantiopure complexes and pro-ligands at the indicated concentrations (µM), for 24 h. Indomethacin (Ind) was used as the reference molecule. Data were plotted as % NO inhibition with respect to LPS-only treatment. Viability assays (blue bars): MTT test was performed under the same experimental conditions of anti-inflammatory activity, in order to verify the effect of the complexes, pro-ligands and indomethacin (Ind) on RAW 264.7 cells viability. Data were plotted as % of cell viability with respect to LPS-only treatment (1 µg/mL). Experiments were performed three times, in triplicate. Columns ± SD are reported. ** *p* > 0.01, *** *p* > 0.001, **** *p* < 0.0001, or not significant (where not indicated), treated vs. LPS only.

Inflammation is an important process, in which the immune system is activated, allowing the protection from infections by eliminating pathogens and promoting repairing phenomena [23]. However, in some cases, the inflammation can become chronic, playing a key role in several diseases, such as cancer, diabetes, autoimmune and neurodegenerative disorders [24]. Moreover, chronic inflammation is characterized by a proliferation of different specialized cells, for instance macrophages, fibroblasts, granulocytes and so on [25]. Nitric oxide (NO) production was demonstrated in murine macrophages [26] and, then, emerged as a major mediator of inflammation. NO is produced by nitric oxide synthases (NOS) isoforms, but under different pro-inflammatory stimuli (cytokines or microbial products) [27] it is massively produced by the inducible NOS (iNOS, NOS2; or type II NOS). iNOS can be also overexpressed in several diseases, producing NO in an unregulated manner and contributing to the disease progression. For these reasons, its inhibition represents a good strategy for the treatment of various diseases related to chronic inflammation, amongst them cancer. The anti-inflammatory activity, in terms of NO production inhibition, of the new synthesized chiral complexes was evaluated by the means of a Griess-based assay. Particularly, the NO production was induced in murine macrophages RAW 264.7 stimulated with bacterial lipopolysaccharide (LPS), then they were exposed to the complexes for 24 h and the released NO was quantified (as detailed in the Section 3). Indomethacin (Ind), a nonsteroidal anti-inflammatory drug (NSAID), was adopted as the positive control, at four different concentrations (see Figure 2), in order to compare the reference drug activity to the studied complexes. Therefore, pro-ligands **P1** and **P2**, as *R* and *S* pure enantiomers, were tested. The obtained outcomes are visible in Figure 2 (panels A and B, **P1** and **P2** series, respectively), where it is possible to notice “at glance” that most of the enantiopure compounds were unable to decrease the NO synthesis, induced by LPS treatment in RAW 264.7 macrophages (see pink bars in the graph), with the exception of **(*R*)-AuL1** and **(*R*)-AuL2**. Particularly, **(*R*)-AuL1** was able to decrease, dose-dependently, the NO production of about 23 and 35% at 5 and 10 µM, respectively, whereas the *S* enantiomer was unable to reduce NO, under the same experimental conditions. As well **(*R*)-AuL2** reduced the NO production, in a dose-dependent manner, of about 10 and 50% at 1 and 5 µM, respectively, but not the ***S*** enantiomer, which was totally inactive at the tested concentrations. Furthermore, none of the pro-ligands was found able to decrease NO production. These results clearly indicate that (i) gold is necessary for the iNOS inhibition, in terms of NO production diminution; (ii) the *R* enantiomers were able to inhibit iNOS (eutomers), whereas *S* enantiomers were totally inactive (dystomers), at least under the adopted experimental conditions; and (iii) the presence of the two chlorine substituents at the carbenic ring enhances the potency (**(*R*)-AuL2** vs. **(*R*)-AuL1**). Moreover, it should also be remarked that both abovementioned eutomers possessed a better ability to decrease the NO synthesis in LPS-stimulated RAW 264.7 macrophages (thus a better anti-inflammatory activity) than Ind, at the adopted concentrations. Indeed, only at 50 µM was Ind able to decrease NO production of about 26%. Finally, viability assays were performed on RAW 264.7 macrophages, recovered after the Griess test. As visible in Figure 2 (panels A and B, see blue bars), the complexes, pro-ligands and Ind did not impact the macrophages viability at the adopted concentrations, compared to the LPS-treated cells, used as the experimental control. Summing up, the obtained outcomes strongly suggested that the stereochemistry of the NHC–gold complexes, together with the substituents at the carbenic ring, are fundamental for the inhibitory activity toward murine iNOS.

### 2.2. Docking Studies

**Figure 3:**
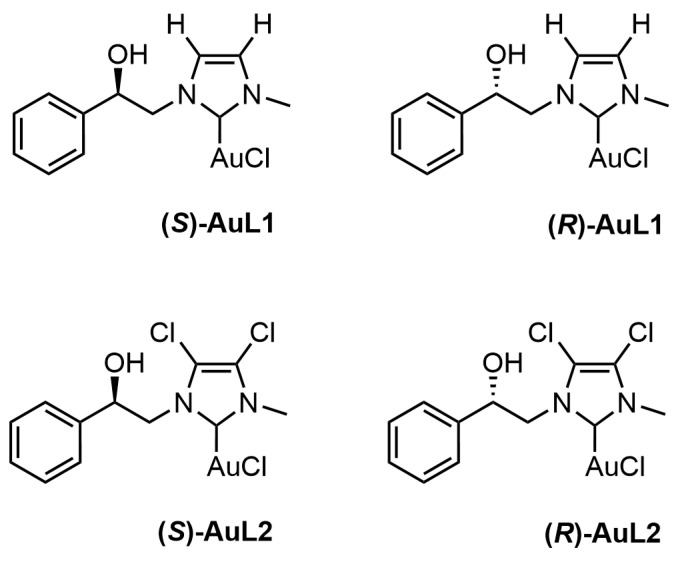
Structures of the docked enantiomerically pure NHC–gold complexes.

Through molecular docking simulations, we evaluated the most probable binding modes of the most interesting complexes to iNOS (Figure 3).

**Figure 4:**
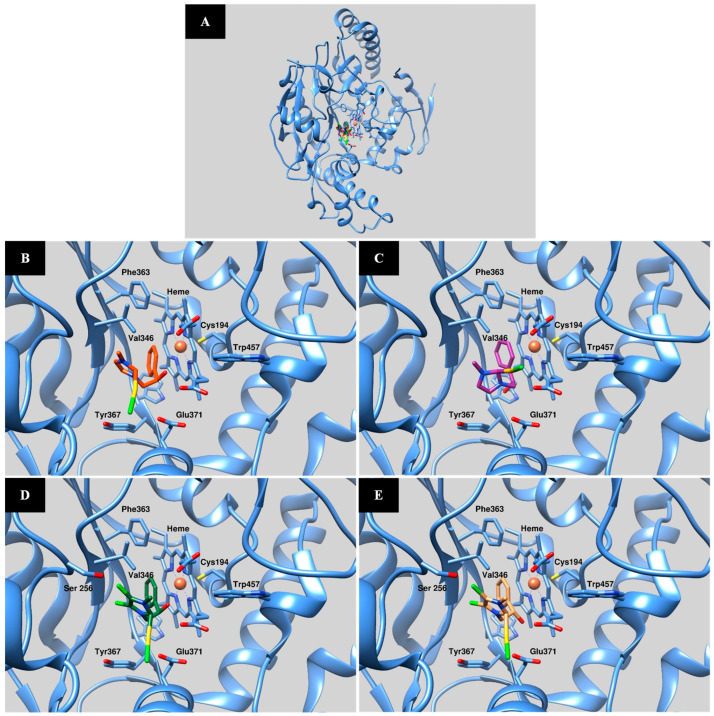
The three-dimensional structure of iNOS (light blue ribbons) in complex with the binding site highlighted by the superposed docked complexes (**A**). (**B**–**E**) illustrate the different binding modes of complexes **(*R*)Au-L1** (orange sticks), **(*S*)-Au-L1** (violet sticks), **(*R*)Au-L2** (green sticks) and **(*S*)Au-L2** (yellow sticks), respectively, and in proximity of the heme moiety. Due to the complexity of the binding site, only some of the residues involved in protein–ligand interactions are drawn as sticks and properly labeled.

We used a “blind-docking approach” for our simulations, a procedure that has been successfully employed by our research group in several previous studies. The goal of these simulations was twofold: to identify the most promising candidate among the tested compounds and to enhance the molecular structure of our compounds. We calculated the binding affinities of the complexes to human iNOS using Autodock. Autodock calculates a possible binding affinity constant (Ki) based on the binding energy between the target protein and the ligand, according to the expression Ki = exp(deltaG/(R*T)). To rank the potential binding modes, we considered the clustering of the simulation results, as discussed in previous works [28]. The best poses obtained for each complex were visually examined to evaluate the quality of the protein interactions. Our docking simulations indicated that our complexes bind iNOS near the protein’s heme group, forming hydrogen bonds and hydrophobic interactions with the protein and its prosthetic group (Figure 4A). All molecules were nearly superimposed on the crystallographic binding site of the molecule *O*-(5-methyl-2-nitrophenyl)-d-tyrosinamide [29]. Results from our docking simulations show that **(*R*)-AuL1** binds the protein forming hydrogen bonds with the heme carboxylate and with protein residue Ser256. The gold atom interacts with Glu371, while the bound chlorine atom forms a halogen bond with Asp376. Hydrophobic interactions with the heme plane and the side chains of residues Ser256, Pro344, Val346, Phe363 and Tyr485 stabilize the binding pose (Figure 4B). Its enantiomer, **(*S*)-AuL1**, instead forms a hydrogen bond with the carboxyl moiety of Trp366 while the gold atom bonds to the heme carboxylate (Figure 4C). **(*R*)-AuL2** forms hydrogen bonds with the heme carboxylate and with the carboxylate and N-peptidic groups of Ala345 and Val346. The ligand is further stabilized by a halogen bond between the gold-bound chlorine atom and the carboxylate of Asp376. The gold atom is bound to the carboxylate group of Glu371 (Figure 4D). Finally, **(*S*)-AuL2** is perfectly superimposed onto its enantiomer **(*R*)-AuL2**, but the opposite configuration causes the loss of its interaction with the heme (Figure 4E).

Binding energies for the four complexes are reported in Table 2.

### 2.3. Anticancer Activity

In the last decade, remarkable results have been obtained in the development of gold– and silver–NHC complexes as anticancer agents, which have been proved to elicit potent and selective anticancer activity and modulate important molecular targets involved in cancer onset and progression [1,30]. However, chirality in organometallic anticancer complexes was investigated mostly for those containing platinum [31], whereas less is known about the gold– and silver–based chiral NHC complexes, even though the obtainment of enantiopure NHC transition metals complexes represents an evolving field in medicinal organometallic chemistry [20]. Conversely, the chiral NHC–gold complexes have found interesting applications in catalysis, as reported in many studies [32]. Considering the strict relationships between cancer and chronic inflammation, and the above-discussed anti-inflammatory properties of the gold enantiopure complexes, they were also tested to evaluate their anticancer properties using two different breast cancer cell lines: the ER-positive MCF-7 and the triple-negative MDA MB-231 cells. The human mammary epithelial cell line MCF-10A was adopted for testing the potential cytotoxicity. These cells were chosen because our research group previously published the anticancer ability of these complexes, as racemic mixture, and studied some of the related molecular mechanisms [13,14]. Thus, herein, we would like to investigate whether the enantiopure complexes could exert a different activity with respect to the racemate. IC_50_ values were calculated for all the complexes and pro-ligands, basing on the viability assays, as detailed in experimental sections and presented in Table 3. Regarding the complexes with the **L1** chiral ligands, it can be noticed that both of the **AuL1** enantiomers impacted the cells’ viability in a similar manner, without any significant difference, with the IC_50_ values being rather close to each other, both in the MCF-7 and MDA-MB.231 cells. Similarly, their cytotoxicity toward the normal breast cells was comparable (**(*R*)-AuL1**and **(*S*)-AuL1** IC_50_ values of 52.9 ± 2.2 and 64.5 ± 3.2 µM, respectively). A difference, instead, was obtained for the **AgL1** complexes, where the ***R*** enantiomer was the most active in both the breast cancer cell lines. A similar trend was recorded against the normal breast cells. Thus, **(R)-AgL1** was able to selectively decrease the viability of the two breast cancer cell lines. As expected, none of the enantiopure **P1** pro-ligands exerted cytotoxicity toward all the cell lines tested, at least above a concentration of 200 µM. Next, the gold **L2** complexes (*R* and *S*), namely **(*R*)-AuL2** and **(*S*)-AuL2**, were found to be very active against both cancer cell lines, but the *R* enantiomer was the most active and selective. Moreover, it did not impact the normal breast cell line viability, with the IC_50_ values being about 19- and 38-fold higher than those calculated for MCF-7 and MDA-MB-231 cells. Conversely, **(*S*)-AuL2** demonstrated a lower anticancer activity and selectivity; indeed, the IC_50_ value toward the normal MCF-10A was found only 1.5-fold higher with respect to those of the breast cancer cells. Regarding the **(*R*)-AgL2**, it was found to exert a fair anticancer activity against both cell lines with a considerable selectivity. **(*S*)-AgL2** decreased the viability of MCF-7 cells better than the *R* enantiomer, with an IC_50_ of 4.0 ± 0.5 µM, but was found inactive against the MDA-MB-231 cells. Furthermore, both AgL2 enantiomers are not cytotoxic. Finally, none of the pro-ligands **(*R*)-** and **(*S*)-P2** exerted any effect. Overall, the obtained outcomes strongly suggest that, for almost all of the studied complexes, the configuration is determinant for the in vitro breast anticancer activity and the selectivity over the normal cells. It should be highlighted that **(*R*)-AuL2** was demonstrated to also possess the best activity in inhibiting the NO production, a feature that, together with the best anticancer activity, makes this complex particularly promising under a pharmacological point of view.

### 2.4. Antibacterial Activity

The new synthesized complexes were also investigated for their potential antibacterial activity. Two different bacterial strains were employed, namely the gram-negative *Escherichia coli* and the gram-positive *Staphylococcus aureus*. All experiments were performed in triplicate. The obtained results, in terms of minimum inhibitory concentration (MIC) and minimum bactericidal concentration (MBC), expressed as µg/mL, are shown in Table 4. Both strains were sensitive to ampicillin; conversely, no inhibition of bacterial growth was observed by DMSO (vehicle) treatment. Our data evidenced that some complexes present a fair antibacterial activity in both bacterial strains, with the ***R*** enantiomer being the most active. Particularly, **(*R*)-AuL1** and **(*R*)-AgL1** showed a MIC of 25 µg/mL against *S. aureus* and *E. coli*, respectively. As for the **L2** series, the complex that had the best MIC was **(*R*)-AgL2** (50 µg/mL against *E. coli*). As expected, the NHC–silver-based complexes had the higher antibacterial activity, in particular both the ***R*** enantiomers. As an exception, **(*R*)-AuL1** was found particularly active toward *S. aureus*. Concerning the determination of the lowest concentration capable of completely killing the two strains of bacteria (MBC), our data showed that all complexes exhibited a MBC equal to or greater than 100 μg/mL. Overall, these data indicate that the synthesized enantiopure complexes possess different biological properties that could act synergistically.

## 3. Materials and Methods

### 3.1. Chemistry

#### 3.1.1. General Methods

All reagents were purchased from Merck Italy (Milan, Italy) and TCI Chemicals (Zwijndrecht, Belgium) and used without purification unless otherwise mentioned.

All solvents were bought by Carlo Erba Reagents srl (Milano, Italy) or Merck Italy (Milan, Italy) and were distilled over appropriate drying agents under nitrogen before use. The synthesis of the metallic complexes was carried out under a nitrogen atmosphere by using Schlenk techniques in the dark. The glassware used was dried in an oven at 120 °C overnight. Deuterated solvents were dried on molecular sieves. ^1^H and ^13^C nuclear magnetic resonance spectra (NMR) were acquired on a Bruker Avance 250 spectrometer (Billerica, MA, USA, 250 MHz for ^1^H; 62.5 MHz for ^13^C) 300 spectrometer (300 MHz for ^1^H; 75 MHz for ^13^C) and a Bruker Avance 400 spectrometer (400 MHz for ^1^H; 100 MHz for ^13^C) operating at 298 K. NMR samples were prepared by dissolving about 10 mg of the compound in 0.5 mL of deuterated solvent (Eurisotop Cambridge Isotope Laboratories, Cambridge, UK). The chemical shifts of ^1^H-NMR and ^13^C-NMR spectra are referenced using the residual proton impurities of the deuterated solvents. ^1^H-NMR were reported relative to DMSO-*d*_6_ δ 2.50 ppm; ^13^C-NMR were reported relative to DMSO-*d*_6_ δ 39.52 ppm. The spectra multiplicities are indicated as follows: singlet (s), doublet (d), triplet (t), multiplet (m), broad (br) and overlapped (o). Coupling constants (J) are quoted in Hertz. ESI-MS measurements of organic compounds were performed on a Waters Quattro Micro triple quadrupole mass spectrometer (Milford, MA, USA) equipped with an electrospray ion source. ESI-FT-ICR measurements of complexes were performed on a Bruker Solaris XR instrument. Optical rotations were measured on a digital polarimeter (JASCO P-2000, Hachioji City, Japan) at a concentration of C = 0.50. A sodium lamp (λ = 589 nm) was used as a light source.

#### 3.1.2. Synthesis of Pro-Ligands, Imidazolium Salts **(*R*)-P1**, **(*S*)-P1**, **(*R*)-P2**, **(*S*)-P2**

Synthesis of the enantiopure imidazolium salts was performed according to literature procedures [22,33], employing enantiopure (*S*)-(−)-styrene oxide or (*R*)-(+)-styrene oxide, purchased by Merck Italy.

**(*S*)- or (*R*)-P1**

Imidazole (1.00 eq) was dissolved in dry acetonitrile (0.03 M), in a round-bottomed flask equipped with a magnetic stirrer, under nitrogen atmosphere; (*S*)-(−)-styrene oxide or (*R*)-(+)-styrene oxide (1.20 eq) were added, followed by K_2_CO_3_ (1.00 eq) then the reaction mixture was stirred at reflux overnight. After 18 h, the mixture was brought to room temperature and filtered to collect the white powders.

(*S*)-1-(2-hydroxy-2-phenylethyl)-1*H*-imidazole (white amorphous powder, 84%).

^1^H NMR (250 MHz, DMSO-*d*_6_, δ ppm): 7.48 (1H, br s, NC*H*N), 7.33 (5H, overlapping signals, Ar-*H*), 7.11 (1H, br s, NC*H*CHN), 6.82 (1H, br s, NCHC*H*N), 5.71 (1H, br s, CHO*H*), 4.81 (1H, m, C*H*OH), 4.12 (1H, dd, *J_syn_* = 4.0 Hz, C*H*HCHOH), 4.02 (1H, dd, *J_anti_* = 7.7 Hz, CH*H*CHOH).

^13^C NMR (62.5 MHz, DMSO-*d*_6_, δ ppm): 142.7 (Ar-*C*), 137.7 (N*C*HN), 128.1 (Ar-*C*H), 127.7 (Ar-*C*H), 127.3 (CH_2_N*C*HCHN), 126.0 (Ar-*C*H), 120.0 (CH_2_NCH*C*HN), 72.1 (*C*H_2_CHOH), 53.5 (CH_2_*C*HOH). ESI-MS (CH_2_Cl_2_): [M + H]^+^ calcd/found (*m*/*z*): [C_11_H_12_N_2_O]^+^ 188.09/189.13.

(*R*)-1-(2-hydroxy-2-phenylethyl)-1*H*-imidazole (white powder, 81%)

^1^H NMR (300 MHz, DMSO-*d*_6_, δ ppm): 7.48 (1H, br s, NC*H*N), 7.34 (5H, overlapping signals, Ar-*H*), 7.11 (1H, br s, NC*H*CHN), 6.82 (1H, br s, NCHC*H*N), 5.70 (1H, br s, CHO*H*), 4.81 (1H, m, C*H*OH), 4.12 (1H, dd, *J_syn_* = 4.2 Hz, C*H*HCHOH), 4.02 (1H, dd, *J_anti_* = 7.9 Hz, CH*H*CHOH).

^13^C NMR (75 MHz, DMSO-*d*_6_, δ ppm): 142.1 (Ar-*C*), 137.2 (N*C*HN), 127.5 (Ar-*C*H), 127.1 (Ar-*C*H), 126.7 (CH_2_N*C*HCHN), 125.4 (Ar-*C*H), 119.5 (CH_2_NCH*C*HN), 71.5 (*C*H_2_CHOH), 52.9 (CH_2_*C*HOH).

ESI-MS (CH_2_Cl_2_): [M + H]^+^ calcd/found (*m*/*z*): [C_11_H_12_N_2_O]^+^ 188.09/189.06.

(*S*)- or (*R*)-1-(2-hydroxy-2-phenylethyl)-1H-imidazole (1.00 eq) were suspended in anhydrous acetonitrile (0.03 M) and brought to reflux for 10 min to allow complete dissolution. Then, the mixture was brought to room temperature and iodomethane (7.00 eq) was added, then it was allowed to stir for a further 5 h at a refluxing temperature. Imidazolinium salts **(*S*)- or (*R*)-P1** and **P2** were collected as white powders after filtration.

**(*S*)-P1**–(*S*)-1-(2-hydroxy-2-phenylethyl)-3-methyl-1*H*-imidazol-3-ium iodide (white amorphous solid, 64%)

^1^H NMR (250 MHz, DMSO-*d*_6_, δ ppm): 9.11 (1H, br s, NC*H*N), 7.70 (2H, br s, NC*H*C*H*N), 7.39 (5H, overlapping signals, Ar-*H*), 5.95 (1H, br s, CHO*H*), 4.94 (1H, m, C*H*OH), 4.43 (1H, m, C*H*HCHOH), 4.22 (1H, m, CH*H*CHOH), 3.88 (3H, br s, C*H_3_*).

^13^C NMR (62.5 MHz, DMSO-*d*_6_, δ ppm): 141.2 (Ar-*C*), 137.0 (N*C*HN), 128.1, 127.7, 127.3, 126.0, 123.0 (aromatic carbons + N*C*H*C*HN), 70.6 (CH_2_*C*HOH), 55.6 (*C*H_2_CHOH), 35.8 (N*C*H_3_).

ESI-MS (CH_2_Cl_2_): calcd/found (*m*/*z*), [C_12_H_15_N_2_O]^+^ 203.11/203.13.

Elemental analysis: Calculated for C_12_H_14_IN_2_O C, 43.79; H, 4.29; I, 38.55; N, 8.51; O, 4.86, Found C, 43.70; H, 4.43; I, 38.43; N, 8.40; O, 4.95.

**(*R*)-P1**–(*R*)-1-(2-hydroxy-2-phenylethyl)-3-methyl-1*H*-imidazol-3-ium iodide (white amorphous solid, 68%)

^1^H NMR (300 MHz, DMSO-*d*_6_, δ ppm): 9.07 (1H, br s, NC*H*N), 7.68 (2H, br s, NC*H*C*H*N), 7.40–7.31 (5H, overlapping signals, Ar-*H*), 5.98 (1H, br s, CHO*H*), 4.92 (1H, m, C*H*OH), 4.40–4.18 (2H, m, C*H_2_*CHOH), 3.87 (3H, br s, C*H*_3_).

^13^C NMR (75 MHz, DMSO-*d*_6_, δ ppm): 140.6 (Ar-*C*), 136.4 (N*C*HN), 127.8, 127.3, 126.4, 125.4, 122.5 (aromatic carbons + N*C*H*C*HN), 70.1 (CH_2_*C*HOH), 55.1 (*C*H_2_CHOH), 35.2 (N*C*H_3_).

ESI-MS (CH_2_Cl_2_): calcd/found (*m*/*z*), [C_12_H_15_N_2_O]^+^ 203.11/203.19.

Elemental analysis: Calculated for C_12_H_14_IN_2_O C, 43.79; H, 4.29; I, 38.55; N, 8.51; O, 4.86, Found C, 43.71; H, 4.40; I, 38.31; N, 8.28; O, 4.70.

**(*S*)- or (*R*)-P2**

4,5-Dichloroimidazole (1.00 eq) was dissolved in dry acetonitrile (0.03 M), in a round-bottomed flask equipped with a magnetic stirrer, under a nitrogen atmosphere; (*S*)-(−)-styrene oxide or (*R*)-(+)-styrene oxide (1.20 eq) was added, followed by K_2_CO_3_ (1.00 eq) then the reaction mixture was stirred at reflux overnight. After 18 h, the mixture was brought to room temperature and filtered to collect the white powders.

(*S*)-1-(2-hydroxy-2-phenylethyl)-4,5-dichloro-1*H*-imidazole

^1^H NMR (250 MHz, DMSO-*d*_6_, δ ppm): 7.69 (1H, br s, NC*H*N), 7.34 (5H, overlapping signals, Ar-*H*), 5.88 (1H, br s, CHO*H*), 4.84 (1H, m, C*H*OH), 4.10–4.06 (2H, overlapping m, C*H_2_*CHOH).

^13^C NMR (62.5 MHz, DMSO-*d*_6_, δ ppm): 141.8 (Ar-*C*), 136.8 (N*C*HN), 128.3, 127.7, 126.0, 123.8, 112.4 (aromatic carbons + N*C*Cl*C*ClN), 70.8 (CH_2_*C*HOH), 52.7 (*C*H_2_CHOH).

ESI-MS (CH_2_Cl_2_): [M + H]^+^ calcd/found (*m*/*z*), [C_11_H_11_Cl_2_N_2_O]^+^ 257.02/258.06.

(*R*)-1-(2-hydroxy-2-phenylethyl)-4,5-dichloro-1*H*-imidazole

^1^H NMR (300 MHz, DMSO-*d*_6_, δ ppm): 7.70 (1H, br s, NC*H*N), 7.34 (5H, overlapping signals, Ar-*H*), 4.84 (1H, m, C*H*OH), 4.14–4.06 (2H, overlapping m, C*H_2_*CHOH).

^13^C NMR (75 MHz, DMSO-*d*_6_, δ ppm): 141.9 (Ar-*C*), 136.7 (N*C*HN), 128.3, 127.5, 126.2, 123.9, 112.8 (aromatic carbons + N*C*Cl*C*ClN), 71.2 (CH_2_*C*HOH), 51.5 (*C*H_2_CHOH).

ESI-MS (CH_2_Cl_2_): [M + H]^+^ calcd/found (*m*/*z*), [C_11_H_11_Cl_2_N_2_O]^+^ 257.02/258.09.

(*S*)- or (*R*)-1-(2-hydroxy-2-phenylethyl)-4,5-dichloro-1H-imidazole (1.00 eq) were suspended in anhydrous acetonitrile (0.03 M), and brought to reflux for 10 min to allow complete dissolution. Then, the mixture was brought to room temperature and iodomethane (7.00 eq) was added, then it was allowed to stir for a further 5 h at a refluxing temperature. Imidazolinium salts **(*S*)-** or **(*R*)-P2** were collected as white powders after filtration.

**(*S*)-P2**–(*S*)-4,5-dichloro-1-(2-hydroxy-2-phenylethyl)-3-methyl-1*H*-imidazol-3-ium iodide (white amorphous solid, 43%)

^1^H NMR (250 MHz, DMSO-*d*_6_, δ ppm): 9.47 (1H, br s, NC*H*N), 7.40 (5H, overlapping signals, Ar-*H*), 6.07 (1H, d, *J* 4.0 Hz, CHO*H*), 4.95 (1H, m, C*H*OH), 4.48–4.25 (2H, overlapping m, C*H_2_*CHOH), 3.90 (3H, s, NC*H_3_*).

^13^C NMR (62.5 MHz, DMSO-*d*_6_, δ ppm): 140.4 (Ar-*C*), 137.2 (N*C*HN), 128.5, 128.1, 126.0, 118.7 (aromatic carbons + N*C*Cl*C*ClN), 69.7 (CH_2_*C*HOH), 54.8 (*C*H_2_CHOH), 35.0 (N*C*H_3_).

ESI-MS (CH_2_Cl_2_): calcd/found (*m*/*z*), [C_12_H_13_Cl_2_N_2_O]^+^ 271.03/271.09.

Elemental analysis: Calculated for C_12_H_12_Cl_2_IN_2_O C, 36.21; H, 3.04; Cl, 17.81; I, 31.88; N, 7.04; O, 4.02, Found C, 36.09; H, 3.07; Cl, 17.93; I, 31.75; N, 7.15; O, 4.11.

**(*R*)-P2**–(*R*)-4,5-dichloro-1-(2-hydroxy-2-phenylethyl)-3-methyl-1*H*-imidazol-3-ium iodide (white amorphous solid, 69%)

^1^H NMR (300 MHz, DMSO-*d*_6_, δ ppm): 9.50 (1H, br s, NC*H*N), 7.40 (5H, overlapping signals, Ar-*H*), 6.06 (1H, br s, CHO*H*), 5.00 (1H, m, C*H*OH), 4.49–4.27 (2H, overlapping m, C*H_2_*CHOH), 3.91 (3H, s, NC*H_3_*).

^13^C NMR (75 MHz, DMSO-*d*_6_, δ ppm): 141.2 (Ar-*C*), 137.1 (N*C*HN), 127.5, 126.8, 125.2, 119.5 (aromatic carbons + N*C*Cl*C*ClN), 71.5 (CH_2_*C*HOH), 53.0 (*C*H_2_CHOH), 34.4 (N*C*H_3_).

ESI-MS (CH_2_Cl_2_): calcd/found (*m*/*z*), [C_12_H_13_Cl_2_N_2_O]^+^ 271.03/271.08.

Elemental analysis: Calculated for C_12_H_12_Cl_2_IN_2_O C, 36.21; H, 3.04; Cl, 17.81; I, 31.88; N, 7.04; O, 4.02, Found C, 36.28; H, 3.13; Cl, 17.74; I, 31.70; N, 7.15; O, 4.14.

#### 3.1.3. Synthesis of Silver(I) Complexes **(*R*)-AgL1**, **(*S*)-AgL1**, **(*R*)-AgL2**, **(*S*)-AgL2**

Pro-ligands **P1**/**P2** (1.00 eq) were dissolved in dry dichloromethane (0.03 M), under stirring in a nitrogen atmosphere. AgNO_3_ (1.00 eq) was added, and the mixture was left to stir at room temperature for 2 h, then K_2_CO_3_ (2.00 eq) was added. The reaction was stirred overnight, then filtered on a Celite plug. The solution was dried under vacuum, and the resulting powder was washed with hexane (3·10 mL) to gain the silver complexes **(*R*)-AgL1, (*S*)-AgL1, (*R*)-AgL2, (*S*)-AgL2** as amorphous, off-white powders.

**(*S*)-AgL1**–(1-((*S*)-2-hydroxy-2-phenylethyl)-3-methyl-2,3-dihydro-1*H*-imidazol-2-yl)silver(I) iodide (off-white powder, 80%)

^1^H NMR (250 MHz, DMSO-*d*_6_, δ ppm): 7.68 (2H, br s, NC*H*C*H*N), 7.41–7.30 (5H, overlapping signals, Ar-*H*), 5.83 (1H, br s, CHO*H*), 4.94 (1H, m, C*H*OH), 4.43–4.16 (2H, overlapping signals, C*H_2_*CHOH), 3.87 (3H, br s, C*H_3_*).

^13^C NMR (62.5 MHz, DMSO-*d*_6_, δ ppm): 181.1 (N*C*N), 142.2 (Ar-*C*), 128.4, 127.7, 127.5, 126.0, 122.8 (aromatic carbons + N*C*H*C*HN), 72.3 (CH_2_*C*HOH), 58.1 (*C*H_2_CHOH), 30.7 (N*C*H_3_).

ESI-MS (CH_3_CN): calcd/found (*m*/*z*), [C_24_H_28_AgN_4_O_2_]^+^ 511.12/511.15.

Elemental analysis: Calculated for C_12_H_15_AgIN_2_O C, 32.90; H, 3.45; Ag, 24.63; I, 28.97; N, 6.40; O, 3.65, Found C, 32.80; H, 3.37; Ag, 24.72; I, 28.85; N, 6.25; O, 3.58.

**(*R*)-AgL1**–(1-((*R*)-2-hydroxy-2-phenylethyl)-3-methyl-2,3-dihydro-1*H*-imidazol-2-yl)silver(I) iodide (off-white powder, 82%)

^1^H NMR (300 MHz, DMSO-*d*_6_, δ ppm): 7.32 (5H, overlapping signals, Ar-*H*), 7.22 (1H, br, CH_3_NCHC*H*N), 6.96 (1H, br, CH_3_NC*H*CHN), 4.85 (1H, m, C*H*OH), 4.53–4.30 (2H, overlapping signals, C*H_2_*CHOH), 3.80 (3H, s, C*H_3_*).

^13^C NMR (75 MHz, DMSO-*d*_6_, δ ppm): 180.3 (N*C*N), 137.0 (Ar-*C*), 128.7, 128.6, 127.3, 122.8, 122.2 (aromatic carbons + N*C*H*C*HN), 76.7 (CH_2_*C*HOH), 56.1(*C*H_2_CHOH), 31.9 (N*C*H_3_).

ESI-MS (CH_3_CN): calcd/found (*m*/*z*), [C_24_H_28_AgN_4_O_2_]^+^ 511.12/511.15 (attributed to the biscarbenic species).

Elemental analysis: Calculated for C_12_H_15_AgIN_2_O C, 32.90; H, 3.45; Ag, 24.63; I, 28.97; N, 6.40; O, 3.65, Found C, 32.88; H, 3.39; Ag, 24.74; I, 28.83; N, 6.27; O, 3.59.

**(*S*)-AgL2**–(4,5-dichloro-1-((*S*)-2-hydroxy-2-phenylethyl)-3-methyl-2,3-dihydro-1*H*-imidazol-2-yl)silver(I) iodide (off-white powder, 41%)

^1^H NMR (250 MHz, DMSO-*d*_6_, δ ppm): 7.46–7.23 (5H, overlapping signals, Ar-*H*), 5.91 (1H, d, CHO*H*), 5.00 (1H, m, C*H*OH), 4.47–4.28 (2H, overlapping m, C*H_2_*CHOH), 3.84 (3H, s, NC*H_3_*).

^13^C NMR (62.5 MHz, DMSO-*d*_6_, δ ppm): 181.2 (N*C*N), 140.4 (Ar-*C*), 128.5, 128.2, 126.3, 118.7 (aromatic carbons + N*C*Cl*C*ClN), 69.8 (CH_2_*C*HOH), 55.1 (*C*H_2_CHOH), 37.6 (N*C*H_3_).

ESI-MS (CH_3_CN): calcd/found (*m*/*z*), [C_24_H_24_AgCl_4_N_4_O_2_]^+^ 646.96/646.91 (attributed to the biscarbenic species).

Elemental analysis: Calculated for C_12_H_13_AgCl_2_IN_2_O C, 28.43; H, 2.59; Ag, 21.28; Cl, 13.99; I, 25.03; N, 5.53; O, 3.16, Found C, 28.39; H, 2.47; Ag, 21.18; Cl, 13.97; I, 25.21; N, 5.48; O, 3.15.

**(*R*)-AgL2**–(4,5-dichloro-1-((*R*)-2-hydroxy-2-phenylethyl)-3-methyl-2,3-dihydro-1*H*-imidazol-2-yl)silver(I) iodide (white powder, 48%)

^1^H NMR (300 MHz, DMSO-*d*_6_, δ ppm): 7.36 (5H, overlapping signals, Ar-*H*), 5.86 (1H, br, CHO*H*), 4.74–4.68 (1H, m, C*H*OH), 4.34–4.17 (2H, overlapping m, C*H_2_*CHOH), 3.80 (3H, s, NC*H_3_*).

^13^C NMR (75 MHz, DMSO-*d*_6_, δ ppm): 180.3 (N*C*N), 137.0 (Ar-*C*), 128.7, 128.6, 127.3, 122.8, 122.2 (aromatic carbons + N*C*H*C*HN), 76.7 (CH_2_*C*HOH), 56.1 (*C*H_2_CHOH), 38.3 (N*C*H_3_).

ESI-MS (CH_3_CN): calcd/found (*m*/*z*), [C_24_H_24_AgCl_4_N_4_O_2_]^+^ 646.96/646.98 (attributed to the biscarbenic species).

Elemental analysis: Calculated for C_12_H_13_AgCl_2_IN_2_O C, 28.43; H, 2.59; Ag, 21.28; Cl, 13.99; I, 25.03; N, 5.53; O, 3.16, Found C, 28.37; H, 2.45; Ag, 21.15; Cl, 13.94; I, 25.23; N, 5.44; O, 3.15.

#### 3.1.4. Synthesis of Gold(I) Complexes **(*R*)-AuL1**, **(*S*)-AuL1**, **(*R*)-AuL2**, **(*S*)-AuL2**

Silver complexes **(*R*)-AgL1, (*S*)-AgL1, (*R*)-AgL2, (*S*)-AgL2** (1.00 eq) were dissolved in dry dichloromethane (0.03 M), under stirring in a nitrogen atmosphere. (CH_3_)_2_SAuCl (1.00 eq) was then added, and the mixture was stirred at room temperature overnight. Filtration on a Celite plug, followed by washing with hexane (3·10 mL) gave the desired gold(I) complexes as yellow powders.

**(*S*)-AuL1**–(1-((*S*)-2-hydroxy-2-phenylethyl)-3-methyl-2,3-dihydro-1*H*-imidazol-2-yl)gold(I) chloride (yellow powder, 52%)

^1^H NMR (250 MHz, DMSO-*d*_6_, δ ppm): 7.56–7.22 (7H, overlapping signals, NC*H*C*H*N and Ar-*H*), 5.83 (1H, br s, CHO*H*), 5.08 (1H, m, C*H*OH), 4.40–4.11 (2H, overlapping signals, C*H_2_*CHOH), 3.74 (3H, br s, C*H_3_*).

^13^C NMR (62.5 MHz, DMSO-*d*_6_, δ ppm): 168.9 (N*C*N), 142.2 (Ar-*C*), 128.4, 127.6, 127.4, 126.0, 121.9 (aromatic carbons + N*C*H*C*HN), 72.6 (CH_2_*C*HOH), 57.7 (*C*H_2_CHOH), 37.8 (N*C*H_3_).

ESI-MS (CH_3_CN): calcd/found (*m*/*z*), [C_24_H_28_AuN_4_O_2_]^+^ 601.18/601.19 (attributed to the biscarbenic species).

Elemental analysis: Calculated for C_12_H_15_AuClN_2_O C, 33.08; H, 3.47; Au, 45.21; Cl, 8.14; N, 6.43; O, 3.67, Found C, 33.11; H, 3.45; Au, 45.18; Cl, 8.17; N, 6.37; O, 3.63.

**(*R*)-AuL1**–(1-((*R*)-2-hydroxy-2-phenylethyl)-3-methyl-2,3-dihydro-1*H*-imidazol-2-yl)gold(I) chloride (off-white powder, 55%)

^1^H NMR (300 MHz, DMSO-*d*_6_, δ ppm): 7.42–7.20 (7H, overlapping signals, NC*H*C*H*N and Ar-*H*), 4.67 (1H, m, C*H*OH), 4.29 (2H, overlapping signals, C*H_2_*CHOH), 3.77 (3H, s, C*H_3_*).

^13^C NMR (75 MHz, DMSO-*d*_6_, δ ppm): 171.8 (N*C*N), 140.9 (Ar-*C*), 129.3, 128.9, 126.5, 122.8, 121.7 (aromatic carbons + N*C*H*C*HN), 74.5 (CH_2_*C*HOH), 58.5 (*C*H_2_CHOH), 38.8 (N*C*H_3_).

ESI-MS (CH_3_CN): calcd/found (*m*/*z*), [C_24_H_28_AuN_4_O_2_]^+^ 601.18/601.11 (attributed to the biscarbenic species).

Elemental analysis: Calculated for C_12_H_15_AuClN_2_O C, 33.08; H, 3.47; Au, 45.21; Cl, 8.14; N, 6.43; O, 3.67, Found C, 33.13; H, 3.43; Au, 45.15; Cl, 8.12; N, 6.39; O, 3.67.

**(*S*)-AuL2**–(4,5-dichloro-1-((*S*)-2-hydroxy-2-phenylethyl)-3-methyl-2,3-dihydro-1*H*-imidazol-2-yl)gold(I) chloride (yellow powder, 56%)

^1^H NMR (250 MHz, DMSO-*d*_6_, δ ppm): 7.39–7.31 (5H, overlapping signals, Ar-*H*), 6.11 (1H, d, CHO*H*), 5.13 (1H, m, C*H*OH), 4.55–4.28 (2H, overlapping m, C*H_2_*CHOH), 3.85 (3H, s, NC*H_3_*).

^13^C NMR (62.5 MHz, DMSO-*d*_6_, δ ppm): 170.2 (N*C*N), 141.2 (Ar-*C*), 128.2, 128.1, 126.1, 116.4 (aromatic carbons + N*C*Cl*C*ClN), 72.1 (CH_2_*C*HOH), 56.5 (*C*H_2_CHOH), 37.1 (N*C*H_3_).

ESI-MS (CH_3_CN): calcd/found (*m*/*z*), [C_24_H_24_AuCl_4_N_4_O_2_]^+^ 737.03/737.08 (attributed to the biscarbenic species).

Elemental analysis: Calculated for C_12_H_13_AuCl_3_N_2_O C, 28.57; H, 2.60; Au, 39.04; Cl, 21.08; N, 5.55; O, 3.17, Found C, 28.55; H, 2.68; Au, 39.08; Cl, 21.03; N, 5.52; O, 3.11.

**(*R*)-AuL2**–(4,5-dichloro-1-((*R*)-2-hydroxy-2-phenylethyl)-3-methyl-2,3-dihydro-1*H*-imidazol-2-yl)gold(I) chloride (yellow powder, 52%)

^1^H NMR (300 MHz, DMSO-*d*_6_, δ ppm): 7.37–7.22 (5H, overlapping signals, Ar-*H*), 4.90 (1H, m, C*H*OH), 4.35–4.19 (2H, overlapping m, C*H_2_*CHOH), 3.80 (3H, s, NC*H_3_*).

^13^C NMR (100 MHz, DMSO-*d*_6_, δ ppm): 171.3 (N*C*N), 141.2 (Ar-*C*), 128.9, 128.8, 127.9, 125.8 (aromatic carbons + N*C*Cl*C*ClN), 76.5 (CH_2_*C*HOH), 56.6 (*C*H_2_CHOH), 37.4 (N*C*H_3_).

ESI-MS (CH_3_CN): calcd/found (*m*/*z*), [C_24_H_24_AuCl_4_N_4_O_2_]^+^ 737.03/737.10 (attributed to the biscarbenic species).

Elemental analysis: Calculated for C_12_H_13_AuCl_3_N_2_O C, 28.57; H, 2.60; Au, 39.04; Cl, 21.08; N, 5.55; O, 3.17, Found C, 28.53; H, 2.61; Au, 39.11; Cl, 21.09; N, 5.50; O, 3.15.

### 3.2. Biology

#### 3.2.1. Cells Culture

All cell lines used for the reported studies were purchased from the American Type Culture Collection (ATCC, Manassas, VA, USA). Human breast cancer cells MCF-7 and MDA-MB-231 were maintained in Dulbecco’s Modified Eagle Medium/Nutrient Mixture F12 Ham (DMEM/F12) supplemented with 5% fetal bovine serum (FBS), 1% L-glutamine and 100 units/mL of penicillin/streptomycin. Human mammary epithelial cells MCF-10A were cultured in DMEM/F12 medium, supplemented with 5% horse serum (HS), 100 units/mL of penicillin/streptomycin, 0.5 mg/mL hydrocortisone, 0.02 µg/mL human epidermal growth factor (EGF), 10 µg/mL insulin and 0.1 mg/mL cholera enterotoxin [34]. The murine macrophages RAW 264.7 were grown in Dulbecco’s Modified Eagle’s Medium (DMEM) high glucose (4.5 g/L), added with 10% fetal bovine serum (FBS), 1% L-glutamine and 100 units/mL of penicillin/streptomycin. Cells were maintained at 37 °C, in a humidified atmosphere containing 5% CO_2_.

#### 3.2.2. MTT Assay

Cell viability was determined using the 3-(4,5-dimethylthiazol-2-y1)-2,5-diphenyltetrazolium bromide assay (MTT, Sigma-Aldrich, Milan, Italy), as in [35]. Cells were treated for 72 h (anticancer activity) or 24 h (anti-inflammatory activity) at different concentrations and the IC_50_ values were obtained using GraphPad Prism 9 software (GraphPad Software, La Jolla, CA, USA).

#### 3.2.3. Anti-Inflammatory Activity

Anti-inflammatory activity was tested adopting the Griess assay on RAW 264.7 murine macrophages, by measuring NO production, as reported by [36]. Specifically, RAW 264.7 cells were plated on 48 multi-wells and treated with the tested molecules at the indicated concentrations; lipopolysaccharide (LPS, Sigma Aldrich, Milan, Italy), at a final concentration of 1 µg/mL, was used to induce inflammation and stimulate NO production. After 24 h, the cell medium and Griess reagent were mixed at a 1:1 ratio and left under agitation for 30 min, at room temperature. Then, absorbance was measured at 540 nm, using a multiplate reader. The absorbance obtained allowed to assess the percentage of NO production compared with the positive control, in which the cells have been treated with LPS.

#### 3.2.4. Minimum Inhibitory Concentration (MIC) and Minimum Bactericidal Concentration (MBC) Determination

The bacterial strains used for MIC and MBC value determinations expressed as µg/mL by the broth dilution method, according to CLSI guidelines, were the following: Gram-negative: *Escherichia coli* (ATCC^®^ 25922TM); Gram-positive: *Staphylococcus aureus* (ATCC^®^ 23235TM). The MIC represents the lowest concentration of compound able to inhibit the visible microbial growth, expressed in μg/mL, whereas the MBC is the lowest concentration of a given compound that can completely kill the bacteria [30]. Bacteria were grown overnight in LB medium (2%), diluted at a density of 4000 colony forming units (CFUs/mL), plated in the 96-well microplates to obtain a total inoculum load of ca. 105 cells/well and then treated with increasing concentrations of the tested complexes (1, 5, 10, 20, 50, 100, 200 μg/mL). Successively, after incubation at 37 °C overnight, the bacterial growth was monitored at a wavelength of 600 nm using a Multiskan spectrophotometer (Multiskan Ex Microplate model; Thermo Scientific, Nyon, Switzerland). MIC or MBC values were obtained by comparing cell density with a positive control (bacterial cells grown in LB medium were added with only the vehicle, DMSO). The results were representative of three independent experiments performed in triplicate and ampicillin (Sigma Aldrich A9393) was used as the control for strain sensitivity.

### 3.3. Docking 

We utilized the three-dimensional structure of human iNOS determined by X-ray crystallography [29] [PDB code 4UX6]. The molecular structures of the four complexes (**(*R*)-AuL1, (*S*)-AuL1, (*R*)-AuL2** and **(*S*)-AuL2**) were constructed and the energy minimized using the program MarvinSketch (ChemAxon Ltd., Budapest, Hungary). To assess the potential binding modes of these four small molecules to human iNOS and their respective binding energies, we employed the software suite Autodock v.4.2.2 [37]. The protocol that we used was adapted from previous studies conducted by our group, which relied on a “blind docking” strategy in all simulations. This approach involved docking the compounds to their receptor without prior knowledge of the binding site. We used the program’s default settings for each run. Protein and ligand preparations were carried out using the ADT graphical interface [38]. Ligands were treated as fully flexible, while the protein target was kept rigid to reduce computational time. Results were analyzed via cluster analysis based on root-mean-square deviation (RMSD) values of each pose relative to the initial geometry. The lowest energy conformation of the most populated cluster was deemed the best candidate. In cases where two or more clusters were nearly equally populated with similar energy distributions, the corresponding ligands were considered suboptimal. Binding modes from our simulations were ranked by binding energy values and subsequently clustered with a 2.0 Å RMSD cutoff. Structural analysis of the lowest energy solutions of each cluster helped identify the putative protein binding site. Ligand binding mode illustrations were created using Chimera 1.17 [39].

### 3.4. Statistical Analysis

Data were analyzed for statistical significance using one-way ANOVA followed by Dunnett’s test, performed by GraphPad Prism 9. Standard deviations (SD) are shown.

## 4. Conclusions

Chirality is a characteristic of many biologically active molecules and it is well known that enantiomers can show different or opposite pharmacological properties, sometimes even being responsible for possible toxic effects. Therefore, the knowledge of the biological and toxicological properties of each enantiomer is desirable and often necessary in the field of medicinal chemistry. In this study, a series of enantiopure NHC–silver and –gold complexes have been synthetized, characterized and evaluated for their biological properties. First, the identified eutomers possess good anti-inflammatory activity, measured as NO production inhibition in LPS-stimulated RAW 264.7 macrophages. In silico simulations suggested some insights for the observed different activity exerted by an enantiomer. Furthermore, the anticancer and antibacterial activities were evaluated, providing an added value to these. In conclusion, in this work the enantiopure NHC–silver and –gold complexes are presented as valid therapeutic options especially for the treatment of inflammation and cancer.

## Figures and Tables

**Table 1:** Specific rotation (${\left[\alpha \right]}_{D}^{25}$) of pro-ligands and of complexes **(*S*)-AgL1**, **(*R*)-AgL1**, **(*S*)-AuL1** and **(*R*)-AuL1**.

Compound	${\left[\alpha \right]}_{D}^{25}$
**(*S*)-P1**	+44.68
**(*S*)-P2**	+73.22
**(*R*)-P1**	−44.79
**(*R*)-P2**	−65.29
**(*S*)-AgL1**	+64.18
**(*R*)-AgL1**	−74.62
**(*S*)-AuL1**	+106.03
**(*R*)-AuL1**	−103.58

**Table 2:** Binding energies and affinities of the four tested Au complexes to iNOS.

Complexes	Binding Energy (Kcal/mol)	Ki (μM)
**(*R*)-AuL1**	−3.7	7.41
**(*S*)-AuL1**	−2.48	7.65
**(*R*)-AuL2**	−0.689	8.41
**(*S*)-AuL2**	−1.32	8.02

**Table 3:** IC_50_ values (µM) of the enantiopure complexes and pro-ligands against breast cancer (MCF-7, MDA-MB-231) and normal (MCF-10A) cell lines. Values are the mean ± standard deviation of three different experiments, performed in triplicate.

		IC_50_ (µM)	
	MCF-7	MDA-MB-231	MCF-10A
**(*R*)-AuL1**	16.0 ± 0.8	15.3 ± 4.5	52.9 ± 2.2
**(*S*)-AuL1**	14.6 ± 1.0	22.6 ± 0.4	64.5 ± 3.2
**(*R*)-AgL1**	14.5 ± 1.2	9.2 ± 0.5	54.8 ± 2.1
**(*S*)-AgL1**	69.8 ± 2.1	>200	>200
**(*R*)-P1**	>200	>200	>200
**(*S*)-P1**	>200	>200	>200
**(*R*)-AuL2**	2.2 ± 0.2	1.2 ± 0.2	38.0 ± 2.2
**(*S*)-AuL2**	10.0 ± 0.5	11.5 ± 0.4	17.2 ± 1.6
**(*R*)-AgL2**	18.4 ± 0.9	6.7 ± 0.7	69.2 ± 2.7
**(*S*)-AgL2**	4.0 ± 0.5	>200	>200
**(*R*)-P2**	>200	>200	>200
**(*S*)-P2**	>200	>200	>200

**Table 4:** MIC and MBC values (expressed as µg/mL) of the new synthesized NHC–gold and –silver complexes and pro-ligands.

M.I.C. [µg/mL] ^[a]^/M.B.C. [µg/mL] ^[b]^		
	*E. coli* ^[c]^	*S. aureus* ^[c]^
**(*R*)-AuL1**	75/>150	25/>150
**(*S*)-AuL1**	75/>150	75/>150
**(*R*)-AgL1**	25/>100	75/>150
**(*S*)-AgL1**	75/>200	50/>100
**(*R*)-P1**	100/>200	75/>150
**(*S*)-P1**	100/>200	75/>150
**(*R*)-AuL2**	75/>150	75/>100
**(*S*)-AuL2**	100/>200	75/>100
**(*R*)-AgL2**	50/>100	75 />150
**(*S*)-AgL2**	75/>200	75/>200
**(*R*)-P2**	75/>200	75/>150
**(*S*)-P2**	75/>150	75/>200

^[a]^ Minimum inhibitory concentration, ^[b]^ minimum bactericidal concentration, ^[c]^ ampicillin-sensitive.

## Data Availability

The data presented in this study are available on request from the corresponding author.

## Supplementary Materials

The following supporting information can be downloaded at: https://www.mdpi.com/article/10.3390/molecules29225262/s1, Figure S1: Circular dichroism (CD) spectra of (A) **(*R*)-AuL1** and (B) **(*S*)-AuL1** (solvent: acetonitrile).
