# Supplementary material for: When Chirality Makes the Difference: The Case of Novel Enantiopure N-Heterocyclic Carbene–Gold and –Silver Complexes
Source: Molecules. 2024 Nov 7;29(22):5262. doi: 10.3390/molecules29225262 (PMC11596155; doi:10.3390/molecules29225262)
Supplement: Supplementary file 1 [file molecules-29-05262-s001.zip › molecules-3248417-supplementary.pdf]

Supplementary materials

When chirality makes the difference: the case of novel enantiopure *N*-heterocyclic carbene-gold and -silver complexes

Maria Marra[1, ‡], Annaluisa Mariconda[2, ‡], Domenico Iacopetta[1], Jessica Ceramella[1, *], Assunta D'Amato[3], Camillo Rosano[4, *], Kateryna Tkachenko[4], Michele Pellegrino[1], Stefano Aquaro[5], Maria Stefania Sinicropi[1, ‖], Pasquale Longo[3, ‖].

[1] Department of Pharmacy, Health and Nutritional Sciences, University of Calabria, Via P. Bucci, 87036 Arcavacata di Rende, Italy; mariamarra1997@gmail.com (M.R.), domenico.iacopetta@unical.it (D.I.), michele.pellegrino@unical.it (M.P.), s.sinicropi@unical.it (M.S.S.)
[2] Department of Science, University of Basilicata, Viale dell'Ateneo Lucano 10, 85100 Potenza, Italy; annaluisa.mariconda@unibas.it (A.M.)
[3] Department of Chemistry and Biology, University of Salerno, Via Giovanni Paolo II, 132, 84084 Fisciano, Italy; asdamato@unisa.it (A.D.); plongo@unisa.it (P.L.)
[4] U.O. Proteomica e Spettrometria di Massa. IRCCS Ospedale Policlinico San Martino, L.go Rosanna Benzi 10, 16132 Genova (I); kateryna.tkachenko@hsanmartino.it (K.T.)
[5] Department of Life, Health and Environmental Sciences, University of L'Aquila, Piazzale Salvatore Tommasi 1, Blocco 11, 67010 L'Aquila - Coppito - Italy; stefano.aquaro@univaq.it (S.A.); stefano.aquaro@unical.it (S.A.).
* Corresponding authors: jessica.ceramella@unical.it (J.C.); camillo.rosano@hsanmartino.it (C.R.)
‡ Co-first authors
‖ Co-senior authors

Figure S1. Circular dichroism (CD) spectra of A) ***(R)*-AuL1** and B) ***(S)*-AuL1** (solvent: acetonitrile).

A)

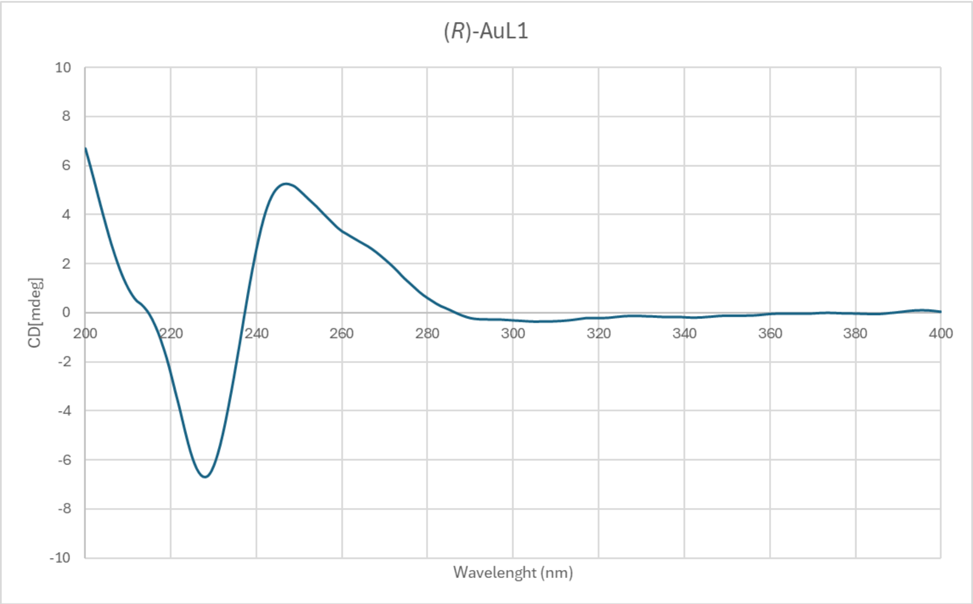

B)

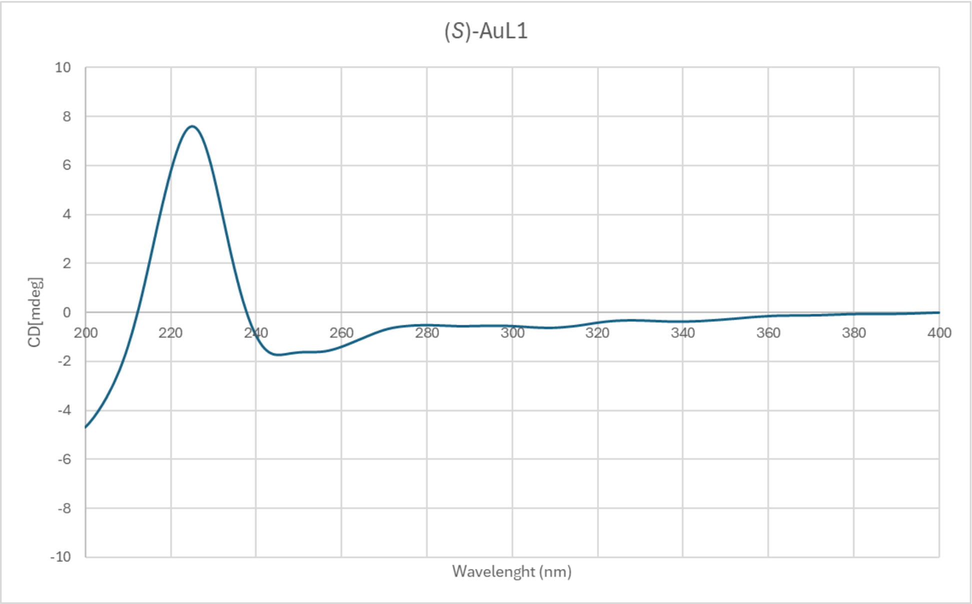

(*S*)-AuL1
CD[mdeg]
10
8
6
4
2
0
-2
-4
-6
-8
-10
200
220
240
260
280
300
320
340
360
380
400
Wavelenght (nm)
